# Novel drug candidates targeting *Toxoplasma gondii* in maternal–fetal interface models

**DOI:** 10.3389/fphar.2025.1673462

**Published:** 2025-12-03

**Authors:** Rafael Martins de Oliveira, Natália Carine Lima dos Santos, Marcos Paulo Oliveira Almeida, Joed Pires De Lima Júnior, Guilherme De Souza, Matheus Carvalho Barbosa, Guilherme Vieira Faria, Marina Paschoalino, Rosiane Nascimento Alves, Angelica Oliveira Gomes, Juliana Quero Reimão, Bellisa Freitas Barbosa, Samuel Cota Teixeira, Eloisa Amália Vieira Ferro

**Affiliations:** 1 Laboratory of Immunophysiology of Reproduction, Institute of Biomedical Science, Universidade Federal de Uberlândia, Uberlândia, Minas Gerais, Brazil; 2 Department of Agricultural and Natural Science, Universidade do Estado de Minas Gerais, Ituiutaba, Minas Gerais, Brazil; 3 Laboratory of Cell Interactions, Institute of Natural and Biological Sciences, Universidade Federal do Triângulo Mineiro, Uberaba, Minas Gerais, Brazil; 4 Laboratory of Preclinical Assays and Research of Alternative Sources of Innovative Therapy for Toxoplasmosis and Other Sicknesses (Lab. PARASITTOS), Department of Morphology and Basic Pathology, Faculdade de Medicina de Jundiaí, Jundiaí, São Paulo, Brazil

**Keywords:** *Toxoplasma gondii*, congenital toxoplasmosis, drug repurposing, Medicines for Malaria Venture, Pathogen Box

## Abstract

**Introduction:**

Toxoplasmosis is a disease caused by the protozoan *Toxoplasma gondii*. Infection during pregnancy can lead to congenital toxoplasmosis, which is associated with severe outcomes such as fetal abnormalities, stillbirths, and miscarriage. Current treatment options for congenital toxoplasmosis have several limitations, including low efficacy in the chronic phase of the disease and concerns about potential fetal toxicity, such as bone marrow suppression and teratogenic effects. Therefore, there is an urgent need for more effective and safer therapeutic alternatives. The Medicines for Malaria Venture (MMV) offers a collection of bioactive compounds with antiparasitic potential for drug repurposing.

**Methods:**

In this study, we evaluated three MMV Pathogen Box compounds—MMV675968, MMV022478, and MMV021013—for their ability to control *T. gondii* infection in human trophoblastic cells (BeWo) and third-trimester placental villous explants. We assessed compound toxicity, effects on the parasite’s lytic cycle (including adhesion and infection), alterations in parasite morphology, and the host immune response through cytokine quantification.

**Results:**

Nontoxic concentrations of all the three compounds irreversibly inhibited parasite proliferation and interfered with early stages of the lytic cycle, including adhesion and infection. Moreover, treated *T. gondii* tachyzoites exhibited membrane disruption, cytoplasmic degradation, and organelle disorganization. The cytokine profile indicated that compound treatment promoted an anti-inflammatory immune response, primarily by reducing IL-8 levels. Importantly, these compounds also effectively controlled *T. gondii* infection in human placental explants without inducing cytotoxicity.

**Conclusion:**

Taken together, our findings support the potential of MMV675968, MMV022478, and MMV021013 as promising drug candidates for the treatment of congenital toxoplasmosis. MMV021013 stands out as the most promising compound, combining high predicted gastrointestinal (GI) absorption and blood–brain barrier (BBB) permeability, with no predicted mutagenic, tumorigenic, irritant, or reproductive effects.

## 1 Introduction

Toxoplasmosis is a globally distributed zoonosis caused by the protozoan parasite *Toxoplasma gondii*, which can infect more than 60% of the population in certain countries (Dubey, 2021). The prevalence of toxoplasmosis is influenced by various factors, such as regional climate and the socioeconomic conditions of the population. Tropical countries, such as those in South America, show higher prevalence rates than European countries, and the lack of access to clean water and adequate sanitation contributes to the spread of the disease (Flegr et al., 2014).

The severity of toxoplasmosis manifestations is directly related to the host’s immune status (Da Silva et al., 2021). In immunocompetent individuals, infection is usually asymptomatic or presents with mild and nonspecific symptoms, such as headache, fever, and myalgia (Al-Malki, 2021). However, in immunocompromised patients, including those with human immunodeficiency virus (HIV), organ transplant recipients, or individuals undergoing chemotherapy, parasite reactivation can lead to severe outcomes, such as encephalitis and chorioretinitis (Walker and Zunt, 2005; Keats Shwab et al., 2018; Smith et al., 2021).

Infection during pregnancy can result in congenital toxoplasmosis, one of the most severe forms of the disease, caused by the transplacental transmission of *T. gondii* tachyzoites. Once across the placenta, the parasites can enter the fetal circulation and tissues, leading to various developmental complications (Rico-Torres et al., 2016; Fallahi et al., 2018). The severity of congenital toxoplasmosis varies depending on the trimester in which infection occurs. First-trimester infections are associated with severe outcomes such as microcephaly, intracranial calcifications, spontaneous abortions, and fetal death (Singh, 2016). Infection during the second trimester can impair neurological and ocular development (McAuley, 2014). In the third trimester, the disease is generally less severe, and most newborns are asymptomatic at birth; however, neurological and visual symptoms may manifest later in childhood or adulthood (Peyron et al., 2019; Schlüter and Barragan, 2019).

The conventional treatment for congenital toxoplasmosis relies on antiparasitic drugs (Fallahi et al., 2018). When maternal infection is confirmed during the first or early second trimester, spiramycin—a macrolide antibiotic—is administered to help prevent transplacental transmission. This drug is recommended only when fetal infection is not yet confirmed as spiramycin does not cross the placenta (Peyron et al., 2017). In cases of confirmed fetal infection, treatment typically involves a combination of sulfadiazine and pyrimethamine, which inhibit enzymes essential for *T. gondii* replication, along with folinic acid supplementation (Doliwa et al., 2013). Although partially effective during the acute phase, these therapies show limited efficacy during chronic infection and are associated with adverse effects on fetal development, including gastrointestinal disturbances, bone marrow suppression, and teratogenicity (Boothroyd and Dubremetz, 2008; Ben-Harari et al., 2017). Additionally, the emergence of drug-resistant strains remains a concern (Meneceur et al., 2008; Montazeri et al., 2018). Therefore, there is an urgent need for new therapeutic strategies that effectively control parasite infection while minimizing host toxicity.

In this context, the repurposing of bioactive compounds originally developed for other pathogens—including viruses, bacteria, and protozoa—has gained attention as a promising approach for toxoplasmosis treatment (Spalenka et al., 2018; Dos Santos B. R. et al., 2023a; Costa et al., 2024). The Medicines for Malaria Venture (MMV) provides open-access libraries containing numerous compounds with antiparasitic potential. Spalenka et al. (2018) conducted preliminary screening assays using one such library, the Pathogen Box, and identified several compounds with strong antiproliferative activity against *T. gondii* in Vero cells, a model of systemic and non-congenital toxoplasmosis. Based on these findings, we selected three compounds—MMV675968, MMV022478, and MMV021013. Given the need for alternative therapies for congenital toxoplasmosis and the promising antiparasitic potential of MMV compounds, the present study aimed to investigate the mechanisms of parasite control mediated by these compounds at the maternal–fetal interface using two distinct experimental models: an *in vitro* model with human trophoblastic BeWo cells and an *ex vivo* model with third-trimester human placental villi.

## 2 Materials and methods

### 2.1 Cell culture and parasite maintenance

Human villous trophoblastic BeWo cells (ATCC, Manassas, VA, United States) were cultured in RPMI 1640 medium (Cultilab, Campinas, SP, Brazil), supplemented with 100U/mL penicillin, 100 μg/mL streptomycin (Sigma Chemical Co., St. Louis, MO, United States), and 5% heat-inactivated fetal bovine serum (FBS) (Cultilab). Cultures were maintained at 37 °C in a humidified 5% CO_2_ atmosphere. In line with protocol number 13/2012 of the Ethics Committee at the Universidade Federal de Uberlândia (MG, Brazil), the use of commercially obtained cell lines does not require ethical approval (Barbosa et al., 2015).

*T. gondii* tachyzoites (2F1 clone of the RH strain, β-galactosidase-expressing) were propagated in BeWo cells in RPMI 1640 medium supplemented with penicillin, streptomycin, and 2% FBS at 37 °C and 5% CO_2_. The 2F1 clone was provided by Professor Dr. Vern Carruthers (University of Michigan, United States) (Barbosa et al., 2015).

### 2.2 Chemical compounds

MMV675968, MMV022478, and MMV021013 from the Pathogen Box were provided by the MMV. Compounds were dissolved in dimethyl sulfoxide (DMSO) to prepare a 10-mM stock solution and then diluted in RPMI 1640 with FBS before use. The final DMSO concentration in the treatments remained below 0.1%, a level nontoxic to BeWo cells (Oliveira et al., 2025).

### 2.3 Human placenta collection

Human placentas (n = 3) were obtained after cesarean delivery from pregnant women (36–40 weeks of gestation) who were seronegative for *T. gondii* and did not present preeclampsia, infectious diseases, heart diseases, or other comorbidities during pregnancy. After collection at the Hospital de Clínicas of UFU (HC-UFU), the acquired placentas were transported to the laboratory, where they were washed with 1× PBS to remove excess blood. Next, dissection was performed to remove the endometrial tissue and fetal membrane. Subsequently, floating terminal placental villi containing five-to-seven free tips per explant (∼10 mm^3^) were collected, placed in 96-well plates (one villus per well), and cultured in RPMI 1640 medium supplemented with 10% FBS and antibiotics (100 U/mL penicillin and 100 μg/mL streptomycin) in a volume of 200 μL per well for 24 h at 37 °C and 5% CO_2_. Informed consent forms were provided for patients to authorize the use of placentas in the experiments. The study was conducted in accordance with the relevant guidelines and regulations, and the experimental protocols were approved by the Ethics Committee of the Federal University of Uberlândia (UFU), MG, Brazil, under approval number 7.407.162.

### 2.4 Cell viability assay

To evaluate the effects of the three selected Pathogen Box drugs on BeWo cell viability, the MTT colorimetric assay (3-[4,5-dimethylthiazol-2-yl]-2,5-diphenyltetrazolium bromide) (Mosmann, 1983) was performed. BeWo cells were cultured at a density of 1 × 10^4^ cells per well in 96-well plates. After cell adhesion, the compounds were added to the plates for 72 h at 37 °C and 5% CO_2._ The three selected compounds were tested at concentrations corresponding to the IC_50_ values previously described by Spalenka et al. (2018). In addition to the IC_50_ concentration, half (½ IC_50_) and double (2× IC_50_) concentrations were also evaluated to explore potential dose-dependent effects. Thus, the tested concentrations were as follows: 0.01 μM, 0.02 μM, or 0.04 μM (MMV675968); 0.14 μM, 0.29 μM, or 0.58 μM (MMV022478); 0.56 μM, 1.12 μM, or 2.24 μM (MMV021013). Cells were also treated with pyrimethamine (PYR; Sigma), a reference drug for toxoplasmosis, at a concentration of 1 μM (Dos Santos B. R. et al., 2023a), whereas treatment with culture medium supplemented with 10% FBS was used as a positive control, considered 100% viability. Next, the supernatant was removed, and the cells were incubated with 100 μL of an MTT solution (5 mg/mL) diluted in culture medium with 10% FBS for 3 h at 37 °C and 5% CO_2_. After this period, 100 μL of 10% sodium dodecyl sulfate (SDS, Sigma) and 50% *N,N*-dimethyl formamide (Sigma) was added to the wells, and the plates were incubated for 18 h at 37 °C and 5% CO_2_. Formazan formation was quantified by measuring absorbance at 570 nm using a microplate photometer (Multiskan, FC, ThermoScientific). The assays were performed at least three independent times, each including a minimum of eight replicates per experimental group.

### 2.5 *T. gondii* proliferation assay

BeWo cells were cultured in 96-well plates at a density of 1 × 10^4^ cells per well in RPMI 1640 medium with 10% FBS for 18 h at 37 °C and 5% CO_2_. After adhesion, cells were infected with *T. gondii* tachyzoites at the proportion of one parasite per cell [multiplicity of infection (MOI) of 1:1] in RPMI 1640 medium with 2% FBS and incubated for 3 h at 37 °C and 5% CO_2_. After this period, the medium was removed, and the wells were washed with 1× PBS to eliminate non-internalized parasites. The cells were then incubated with the following compounds separately: MMV675968 (0.01 μM, 0.02 μM, or 0.04 μM), MMV022478 (0.14 μM, 0.29 μM, or 0.58 μM), and MMV021013 (0.56 μM, 1.12 μM, or 2.24 μM). Additionally, treatment with PYR (1 μM) was used as a classical treatment against toxoplasmosis, whereas culture medium supplemented with 10% FBS was used as a positive proliferation control. After 72 h of treatment, the β-galactosidase assay was performed. In brief, cells were lysed with 100 μL of RIPA buffer [50 mmol/L Tris-HCl, 150 mmol/L NaCl, 1% (v/v) Triton X-100, 1% (w/v) sodium deoxycholate, and 0.1% (w/v) SDS; pH 7.5] for 15 min and then incubated with 160 μL of assay buffer (100 mM PBS, 102 mM β-mercaptoethanol, and 9 mM MgCl_2_) and 40 μL of 6.25 mM chlorophenol red-D-galactopyranoside (CPRG). β-Galactosidase activity was measured at 570 nm using a spectrophotometer, and intracellular *T. gondii* proliferation was determined based on the number of parasites obtained according to a standard curve containing free tachyzoites (1 × 10^6^ to 1.56 × 10^4^). Data were expressed as the percentage (%) of *T. gondii* proliferation (Teixeira et al., 2020). The assays were performed at least three independent times, each including a minimum of eight replicates per experimental group.

### 2.6 Reversibility assay

BeWo cells were simultaneously cultured in two 96-well plates and infected with *T. gondii* tachyzoites at an MOI of 1:1 for 3 h at 37 °C and 5% CO_2_, as described above. Next, the wells were washed with 1× PBS to remove non-internalized parasites, and the cells were incubated for 72 h with the following compounds separately: MMV675968 (0.02 μM), MMV022478 (0.29 μM), or MMV021013 (1.12 μM). Cells were also treated with PYR (1 μM), whereas culture medium supplemented with 10% FBS was used as a proliferation control. After 72 h of treatment, the β-galactosidase assay was performed on one of the plates to assess the *T. gondii* intracellular proliferation. In parallel, the medium in the wells of the second plate was removed and replaced with the fresh culture medium for an additional 24 h. After this period, the β-galactosidase assay was performed to quantify the number of parasites. β-Galactosidase activity was measured at 570 nm using a spectrophotometer, and *T. gondii* intracellular proliferation was determined based on the number of parasites obtained according to a standard curve containing free tachyzoites. Data were expressed as the percentage (%) of *T. gondii* proliferation. The assays were performed at least three independent times, each including a minimum of eight replicates per experimental group.

### 2.7 Infection and intracellular proliferation assays with pretreated *T. gondii* tachyzoites

BeWo cells were seeded in 96-well plates at a density of 3 × 10^4^ cells per well for infection assays or 1 × 10^4^ cells per well for intracellular proliferation assays and cultured for 18 h at 37 °C and 5% CO_2_. For the infection/proliferation assays, tachyzoites were incubated for 1 h at 37 °C with the following compounds: MMV675968 (0.02 μM), MMV022478 (0.29 μM), or MMV021013 (1.12 μM). Parasites were also pretreated with PYR (1 μM) as a death control, whereas culture medium supplemented with 10% FBS was used as a proliferation control. After the treatment period, the parasites were centrifuged (425 × g/7 min) and washed with 1× PBS to remove the treatments. For the first set of experiments, the cells were infected at an MOI of 1:1 with *T. gondii* tachyzoites. After 3 h of infection, the wells were washed with 1× PBS to remove extracellular parasites, and the β-galactosidase assay was performed to assess infection.

In the second set of experiments, the cells were infected at an MOI of 1:1 with *T. gondii* tachyzoites as described above. After 72 h of infection, the β-galactosidase assay was performed to assess proliferation. *T. gondii* infection or proliferation was determined based on the number of parasites obtained according to a standard curve containing free tachyzoites. Data were expressed as the percentage (%) of *T. gondii* infection or proliferation. The assays were performed at least three independent times, each including a minimum of eight replicates per experimental group.

### 2.8 Infection assay with pretreated BeWo cells

BeWo cells were cultured in 96-well plates at a density of 1 × 10^4^ cells per well in RPMI 1640 medium with 10% FBS for 18 h at 37 °C and 5% CO_2_. After adhesion, the cells were treated for 72 h with the following compounds: MMV675968 (0.02 μM), MMV022478 (0.29 μM), or MMV021013 (1.12 μM). After the treatment period, the cells were infected at an MOI of 1:1 with *T. gondii* tachyzoites for 3 h. The wells were then washed with 1× PBS to remove extracellular parasites, and the β-galactosidase assay was performed to assess infection. β-Galactosidase activity was measured at 570 nm using a spectrophotometer, and the *T. gondii* infection was determined based on the number of parasites obtained according to a standard curve containing free tachyzoites. Data were expressed as the percentage (%) of *T. gondii* infection. The assays were performed at least three independent times, each including a minimum of eight replicates per experimental group.

### 2.9 Cell adhesion assay

To investigate whether the three selected compounds are able to impair parasite adhesion to host cells, we proceed with an adhesion assay, as previously described (Teixeira et al., 2020). BeWo cells were seeded on 13-mm coverslips in 24-well plates at a density of 1 × 10^5^ in RPMI 1640 medium with 10% FBS for 18 h at 37 °C and 5% CO_2_. Next, the cells were fixed with 4% paraformaldehyde (PFA) for 30 min at room temperature and washed three times with 1× PBS.

In parallel, *T. gondii* tachyzoites were pretreated for 1 h with the following: MMV675968 (0.02 μM), MMV022478 (0.29 μM), MMV021013 (1.12 μM), PYR (1 μM), or culture medium supplemented with 10% FBS (control group), for 1 h at 37 °C and 5% CO_2_. The pretreated parasites were rinsed and resuspended in a treatment-free culture medium before incubation with previously fixed BeWo cells for 3 h at 37 °C and 5% CO_2_. After that, we removed non-adherent parasites with 1× PBS, and the adhered parasites were fixed as mentioned above.

The coverslips were gently washed once with PBS, incubated with the rabbit polyclonal primary anti-*T. gondii* antibody (Abcam #20530; Waltham, MA, United States) (diluted 1: 500 in PGN-0.01% saponin solution) for 1 h, and rinsed three times with 1× PBS. Next, coverslips were incubated with Alexa Fluor 488-conjugated anti-rabbit IgG (diluted 1:500 in PGN-0.01% saponin solution) (Invitrogen, United States #A11008; Waltham, MA, United States), tetramethylrhodamine isothiocyanate (TRITC)-conjugated phalloidin (diluted 1:50 in PGN + saponin), and the cell nucleus marker TO-PRO-3 Iodide (Life Technologies, Waltham, MA, United States) (diluted 1:500 in PGN + saponin) for an additional 1 h in the dark at room temperature to label *T. gondii* tachyzoites, F-actin, and nuclei, respectively. The coverslips were washed three times with 1× PBS and mounted on glass slides. Afterward, the samples were analyzed using a confocal fluorescence microscopy (Zeiss, LSM 510 Meta, Germany) with an inverted microscope (Zeiss Axiovert 200 M), with excitation wavelengths at 488 nm (Argon laser) for Alexa Fluor 488, 543 nm HeNe laser for TRITC, and 633 nm (HeNe laser) for TO-PRO-3. Emission detection wavelengths were set to 519 nm for Alexa Fluor 488, 570 nm for TRITC, and 661 nm for TO-PRO-3. Immunofluorescence images were acquired at ×40 magnification. The results were expressed by the ratio (parasites/nucleus) in a total of 20 fields chosen randomly. The assays were performed at least three independent times, each including a minimum of eight replicates per experimental group.

### 2.10 Scanning electron microscopy (SEM)

*T. gondii* tachyzoites were pretreated for 1 h with MMV675968 (0.02 μM), MMV022478 (0.29 μM), MMV021013 (1.12 μM), PYR (1 μM), or culture medium supplemented with 10% FBS, for 1 h at 37 °C and 5% CO_2_. After the treatment period, the parasites were centrifuged (2000 rpm/7 min) and washed with cacodylate buffer. Then, the parasites were washed again and fixed in Karnovsky’s buffer for 2 h at 4 °C. After fixation, the samples were washed twice with cacodylate buffer and post-fixed in 1% osmium tetroxide (OsO_4_) for 1 h. Subsequently, the samples were centrifuged to discard OsO_4_, washed twice with cacodylate buffer, and placed on glass coverslips, which were left to dry for 18 h at room temperature. The following day, the samples underwent a dehydration process, in which they were immersed in solutions with increasing concentrations of alcohol (50%, 70%, 80%, 90%, 95%, and 100%) for 5 min in each concentration. Finally, a thin gold layer was applied to the samples, which were analyzed under a scanning electron microscope (Tescan Vega-3 LMU). Scanning electron microscopy (SEM) images were acquired at an accelerating voltage of 5 kV with a magnification of ×10,000. Brightness and contrast adjustments were applied uniformly across all images. For each experimental group, at least 20 fields from different replicates were analyzed to assess the ultrastructural effects of the compounds.

### 2.11 Transmission electron microscopy (TEM)

BeWo cells (5 × 10^5^ cells/well) were seeded in a 6-well plate for 18 h at 37 °C and 5% CO_2_. Afterward, the cells were infected with *T. gondii* tachyzoites (MOI: 1:1) for 3 h. Next, the medium was removed, and the cells were washed with 1× PBS to eliminate non-internalized parasites. The cells were then incubated for 72 h with the following: MMV675968 (0.02 μM), MMV022478 (0.29 μM), MMV021013 (1.12 μM), PYR (1 μM), or culture medium only (untreated group), for 72 h at 37 °C and 5% CO_2_. The cells were harvested and fixed with Karnovsky solution containing 2% PFA and glutaraldehyde in a 0.1 M sodium cacodylate buffer (pH 7.4) at 4 °C for 24 h. Following fixation, the samples were post-fixed with 1% OsO_4_ for 2 h. After this period, the cells were centrifuged to discard OsO_4_, embedded in 5% agar, and stored at 4 °C for 48 h. Subsequently, the samples were removed from the agar and underwent a dehydration process, being immersed in alcoholic solutions of increasing concentrations (50%, 70%, 80%, 90%, and 95%) for 5 min each, followed by three immersions in 100% ethanol (10 min each) and three immersions in propylene oxide solution (10 min each). After dehydration, a propylene oxide/resin solution (2:1 ratio) was added to the samples for 18 h at room temperature. The next day, the propylene oxide/resin solution (2:1) was replaced with a 1:1 ratio solution, and the samples were stored in an incubator for 18 h at 37 °C. On the following day, the 1:1 solution was removed, and the samples were placed in molds for embedding in pure resin and maintained in an incubator at 60 °C for 3 days. After 3 days, the samples were contrasted with uranyl acetate (40 min/30 °C) and lead citrate + NaOH (20 min at room temperature) and finally analyzed under a transmission electron microscope (Hitachi, TM 3000). Transmission electron microscopy (TEM) images were acquired at an accelerating voltage of 80 kV with magnifications ranging from ×2,000 to ×6,000. Brightness and contrast adjustments were uniformly applied to all samples. For each experimental group, at least 20 fields from different replicates were analyzed to assess the ultrastructural effects of the compounds.

### 2.12 Evaluation of reactive oxygen species (ROS) production

To assess intracellular reactive oxygen species (ROS) production, BeWo cells (1 × 10^4^/well) were cultured in a black 96-well microplate with clear bottoms (Costar REF# 3603, New York, United States of America) in RPMI 1640 medium supplemented with 10% FBS for 18 h at 37 °C and 5% CO_2_. After adhesion, the cells were either infected or not with *T. gondii* (MOI: 1:1) for 3 h and subsequently treated or not for 72 h with the following compounds: MMV675968 (0.02 μM), MMV022478 (0.29 μM), MMV021013 (1.12 μM), or PYR (1 μM). After treatment, the supernatants were collected and stored at −80 °C for further cytokine quantification, and the cells were incubated with 100 μL of the probe 2′,7′-dichlorodihydrofluorescein diacetate (H_2_DCF-DA, Invitrogen, catalog number: D399) at a concentration of 10 μM diluted in 1× PBS with 10% FBS for 45 min at 37 °C and 5% CO_2_ in the dark. As a positive control for ROS induction, cells were treated with 3.5% hydrogen peroxide (H_2_O_2_) for 30 min. Following incubation, the probe was removed, and 1× PBS was added to the wells for fluorescence measurement. The 2′,7′-dichlorofluorescein (DCF) fluorescence intensity was detected immediately using a multi-well scanning spectrophotometer (Versa Max ELISA Microplate Reader, Molecular Devices, Sunnyvale, CA, EUA), with excitation and emission wavelengths of 488 nm and 522 nm, respectively. Data were presented as median fluoresce intensity (MFI). The assays were performed at least three independent times, each including a minimum of eight replicates per experimental group.

### 2.13 Viability assays of human villous explants

As previously described, chorionic villi were collected from three term placentas provided by independent donors, cultured in 96-well plates in RPMI 1640 medium with 10% FBS, and then treated or not with the following: MMV675968 (0.02 μM), MMV022478 (0.29 μM), and MMV021013 (1.12 μM), or with PYR (1 μM) for 72 h at 37 °C and 5% CO_2_. After the treatment period, the supernatant from the wells was collected for LDH release analysis, and the explants were incubated for 3 h with 20 μL of MTT (5 mg/mL) + 180 μL of culture medium. Following MTT incubation, formazan crystals were solubilized with 100 μL of 10% SDS and 50% *N,N*-dimethyl formamide for 18 h at 37 °C and 5% CO_2_. Optical density was measured using a microplate reader at a wavelength of 570 nm. The results were expressed as the percentage of viable villi (% villous viability) relative to untreated villi (100% viability). LDH concentration was measured according to the manufacturer’s instructions with minor modifications (LDH UV, Bioclin, Belo Horizonte, MG, Brazil | Ref K014-2). This assay is based on the consumption and reduction of NADH absorption at 340 nm, measured using a microplate reader (Versa Max ELISA Microplate Reader, Molecular Devices, Sunnyvale, CA, United States). LDH release into the culture medium was expressed in U/L of LDH enzymatic activity and used as a marker of tissue integrity. Additionally, to validate the tissue viability of the treated placental explants, the morphological evaluation was carried out. For this purpose, formalin-fixed and paraffin-embedded tissue sections of the placental explants were subjected to hematoxylin–eosin staining and digitalized using an Aperio VERSA 200 BF & Fluorescence scanner (Leica VERSA 200 BF & Fluorescence; Leica Biosystems, Germany). Afterward, representative fields of the tissue sections, from the different experimental conditions, were captured at ×10 of magnification with 200 µm scale bar, using Aperio ImageScope version 12.4.6.5003 software (Leica Biosystems). For each experimental group, eight placental villi (n = 8) were analyzed per placenta, and the assays were repeated independently.

### 2.14 *T. gondii* proliferation in human placental explants

To evaluate the *T. gondii* intracellular proliferation in human placental explants, chorionic villi were collected from three term placentas provided by independent donors, cultured in 96-well plates in RPMI 1640 medium with 10% FBS, and then infected with *T. gondii* tachyzoites (1 × 10^6^ parasites/200 μL/well) and incubated for an additional 24 h at 37 °C and 5% CO_2_. After that, explants were treated or not with the following: MMV675968 (0.02 μM), MMV022478 (0.29 μM), and MMV021013 (1.12 μM), or with PYR (1 μM) for 72 h at 37 °C and 5% CO_2_. Next, culture supernatants were collected and stored at −80 °C for later quantification of cytokine levels. Additionally, explant samples were collected and stored at −80 °C for subsequent analysis of protein content using the Bradford reagent (Sigma) and assessment of *T. gondii* intracellular proliferation via the β-galactosidase assay, as previously described. In brief, the villi were collected and transferred to 1.5 mL tubes containing 150 μL of RIPA buffer [50 mM Tris-HCl, 150 mM NaCl, 1% Triton X-100, 1% (w/v) sodium deoxycholate, and 0.1% (w/v) sodium dodecyl sulfate (SDS); pH 7.5] supplemented with a protease inhibitor cocktail (Complete, Roche Diagnostic, Mannheim, Germany), where they were macerated and homogenized for protein extraction. The homogenate was then centrifuged at 14.000 rpm for 15 min at 4 °C, and the supernatant was collected to measure total protein concentration (μg/mL) using the Bradford method. To determine the proliferation of *T. gondii* in the villi, 20 μL of each sample were used for the β-galactosidase assay, performed as previously described. The number of parasites was normalized according to the protein concentration of each villus and represented as a percentage of *T. gondii* intracellular proliferation, with the average number of tachyzoites in the control group (infected but untreated villi) set as 100% proliferation. For each experimental group, eight placental villi (n = 8) were analyzed per placenta, and the assays were repeated independently.

### 2.15 Cytokine quantification

The levels of human cytokines IL-4, IL-6, IL-8, IL-10, TNF-α, and MIF released into the culture supernatants of BeWo cells or placental villi, either infected or not with *T. gondii* and treated or not with the following compounds: MMV675968 (0.02 μM), MMV022478 (0.29 μM), MMV021013 (1.12 μM), or PYR (1 μM), were measured using an ELISA assay following the manufacturers’ instructions (BD Bioscience, San Diego, CA, United States; R&D Systems, Minneapolis, MN, United States). In brief, the plates were incubated for 18 h at 4 °C with monoclonal anti-IL-4, anti-IL-6, anti-IL-8, anti-IL-10, and anti-TNF-α antibodies (BD Biosciences), along with anti-MIF antibodies (R&D Systems). The next day, the plates were washed with PBS containing 0.05% Tween, and nonspecific binding sites were blocked using PBS with 10% FBS for IL-4, IL-6, IL-8, IL-10, and TNF-α, along with PBS with 1% bovine serum albumin (BSA) for MIF for 1 h at room temperature. After that, the plates were washed again, and the samples and standard curves were added, followed by a 2-h incubation period at room temperature. Subsequently, the plates were washed again and incubated with detection antibodies: streptavidin–peroxidase-conjugated antibodies for IL-4, IL-6, IL-8, IL-10, and TNF-α for 1 h, and anti-MIF antibody for 2 h. For MIF, additional washes were performed, followed by the addition of peroxidase-conjugated streptavidin for 20 min. Finally, 3,3′,5,5′-tetramethylbenzidine (TMB) was added to the plates for immunocomplex detection. The reaction was stopped with H_2_SO_4_, and plate readings were performed at 450 nm using a microplate reader (Titertek Multiskan Plus, Flow Laboratories). Data were expressed in pg/mL according to a standard curve of each cytokine for BeWo cells, whereas for placental explants, cytokine concentrations were normalized using a ratio between cytokine production (pg/mL) and its corresponding total protein content (μg/mL) of each sample, with the levels being demonstrated as pg/mg of tissue. The detection limits were as follows: IL-6 (4.7 pg/mL), IL-8 (6.25 pg/mL), MIF (93.8 pg/mL), IL-4, IL-10, and TNF (all 7.8 pg/mL). The assays were performed at least three independent times, each including a minimum of eight replicates per experimental group.

### 2.16 Pharmacokinetic and toxicity *in silico* predictions

The pharmacokinetic and toxicity profiles of the MMV compounds and PYR were predicted through computational analysis to identify the compounds with favorable characteristics. ADME parameters, including gastrointestinal (GI) absorption and blood–brain barrier (BBB) permeability, were predicted using the SwissADME platform (http://www.swissadme.ch) (Daina et al., 2017). Toxicological properties—such as mutagenicity, tumorigenicity, and reproductive toxicity—were predicted using OSIRIS Property Explorer (Sander et al., 2009).

### 2.17 Statistical analysis

Statistical analysis was performed using GraphPad Prism 8.01 software. Data were expressed as mean ± standard error of the mean (SEM), and differences between groups were evaluated using a one-way ANOVA test with Sidak’s multiple-comparison *post hoc* test for parametric data or the Kruskal-Wallis test with Dunn’s multiple-comparison *post hoc* test for nonparametric data. Statistical differences were considered significant when *p* < 0.05.

## 3 Results

### 3.1 Non-cytotoxic concentrations of the three selected Pathogen Box compounds irreversibly inhibit *T. gondii* intracellular proliferation

**FIGURE 1:**
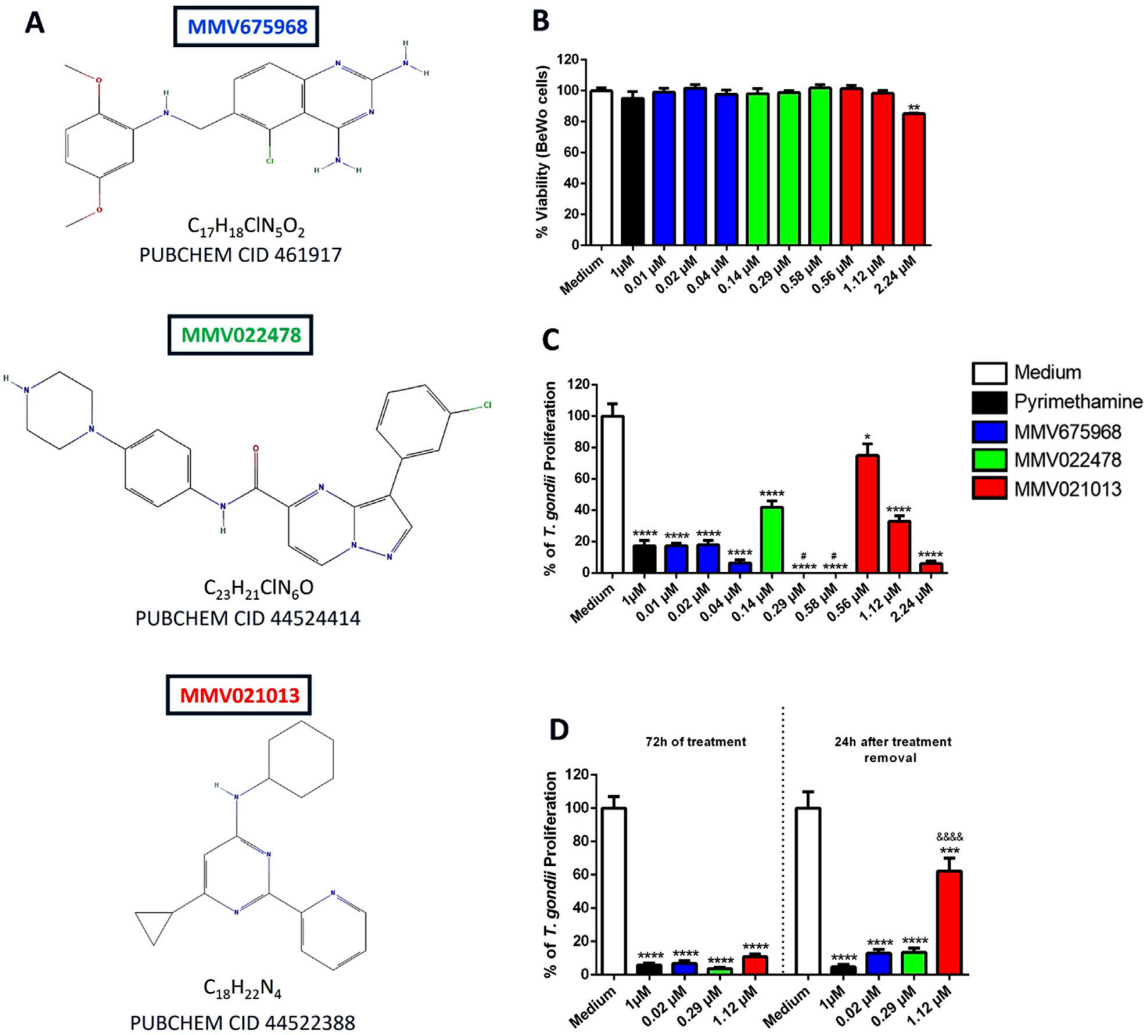
MMV675968, MMV022478, and MMV021013 are nontoxic at low concentrations and irreversibly control *T. gondii* proliferation in BeWo cells. **(A)** Chemical structures of MMV675968 (C_17_H_18_ClN_5_O_2_), MMV022478 (C_23_H_21_ClN_6_O), and MMV021013 (C_18_H_22_N_4_). **(B)** BeWo cells were exposed for 72 h to different concentrations of MMV675968 (0.01, 0.02, and 0.04 μM), MMV022478 (0.14, 0.29, and 0.58 μM), and MMV021013 (0.56, 1.12, and 2.24 μM) or PYR (1 μM). Untreated cells, exposed only to the culture medium, served as the positive control for cell viability and were considered 100% viable. Cell viability was assessed using the MTT assay and expressed as the mean percentage (%) of viable BeWo cells. The results were normalized to the untreated control **(C)** *T. gondii*-infected BeWo cells were exposed for 72 h to MMV675968, MMV022478, MMV021013, and PYR or culture medium only (considered 100% parasite proliferation) as described above. Intracellular parasite proliferation was assessed using the β-galactosidase assay and expressed as a percentage change relative to the control (% *T. gondii* proliferation). **(D)** Infected BeWo cells were treated for 72 h with MMV675968 (0.02 μM), MMV022478 (0.29 μM), MMV021013 (1.12 μM), and PYR (1 μM) or culture medium only; in parallel, the same treatments were removed from infected cells, which were then maintained in a treatment-free medium for an additional 24 h. Under both experimental conditions, intracellular parasite proliferation was assessed using the β-galactosidase assay. The reversibility assay evaluates the parasites’ capacity to recover from treatment and regain infectivity. Data are shown as means ± standard error of the mean (SEM). ^*^ *p* < 0.05, ^**^ *p* < 0.01, ^***^ *p* < 0.001, and ^****^ *p* < 0.0001 indicate comparison between untreated and treated cells. ^#^ *p* < 0.05 indicates comparison to PYR-treated cells; ^&&&&^ *p* < 0.0001 indicates comparison between MMV021013-treated cells after 72 h of treatment and MMV021013-treated cells 24 h after treatment removal. Significant differences were analyzed using the one-way ANOVA test with Sidak’s multiple-comparison *post hoc* test. Differences were considered statistically significant when *p* < 0.05. Data are representative of at least three independent experiments, each performed with a minimum of eight replicates per group.

Initially, the cytotoxicity of three compounds from MMV’s Pathogen Box – MMV675968, MMV022478, and MMV021013 (Figure 1A) – was evaluated in BeWo cells at three different concentrations previously reported by Spalenka et al. (2018), after 72 h of treatment. Cell viability was assessed using the MTT assay. The results showed that only MMV021013 exhibited cytotoxicity at the highest tested concentration (2.24 μM), with a significant reduction in viability compared to that in the untreated group (^**^ *p* < 0.01). Treatment with PYR did not significantly differ from the control (RPMI medium only) (Figure 1B).

To evaluate the compounds’ ability to inhibit *T. gondii* proliferation in BeWo cells, a β-galactosidase assay was performed after 72 h of treatment. All tested concentrations significantly reduced parasite proliferation compared to the control group (^*^ *p* < 0.05; ^****^ *p* < 0.0001). As expected, treatment with PYR (1 μM) significantly reduced parasite proliferation after 72 h (^****^ *p* < 0.0001). Notably, MMV022478 at 0.29 and 0.58 μM demonstrated greater potency than PYR (^#^ *p* < 0.05; Figure 1C). For subsequent experiments, we selected the intermediate concentrations of MMV675968 (0.02 μM), MMV022478 (0.29 μM), and MMV021013 (1.12 μM) as they were nontoxic to BeWo cells and effectively inhibited intracellular *T. gondii* proliferation, ensuring both host cell viability and antiparasitic efficacy.

To assess whether the antiproliferative effects of the compounds were reversible, *T. gondii*-infected BeWo cells were treated for 72 h. Subsequently, the compounds were removed, and the cells were incubated in fresh medium for an additional 24 h. The β-galactosidase assay was used to evaluate intracellular parasite proliferation both at the end of the 72-h treatment and after the 24-h drug-free period. Our data showed that MMV675968 (0.02 μM), MMV022478 (0.29 μM), MMV021013 (1.12 μM), and PYR (1 μM) significantly inhibited parasite proliferation compared to the control group (^****^ *p* < 0.0001; Figure 1D). Importantly, the antiproliferative effects of MMV675968, MMV022478, and PYR were maintained after compound removal. In contrast, partial recovery of parasite proliferation was observed in the group treated with MMV021013, as indicated by a significant increase in β-galactosidase activity 24 h after compound removal compared to the corresponding 72-h treatment condition (^&&&&^ *p* < 0.0001; Figure 1D).

### 3.2 Pre-treatment with MMV675968, MMV022478, and MMV021013 affects early stages of *T. gondii* infection in BeWo cells

To investigate the potential targets of the compounds in controlling parasitism in BeWo cells, we first evaluated their direct action on the parasite. *T. gondii* tachyzoites were pretreated for 1 h with MMV675968 (0.02 μM), MMV022478 (0.29 μM), or MMV021013 (1.12 μM). This 1-h pretreatment was selected as the minimal exposure time sufficient to induce detectable effects on tachyzoites while preserving viability. Following pretreatment, the parasites were used to infect BeWo cells for 3 h (to assess infection) or 72 h (to assess proliferation). After each infection period, the β-galactosidase assay was performed.

**FIGURE 2:**
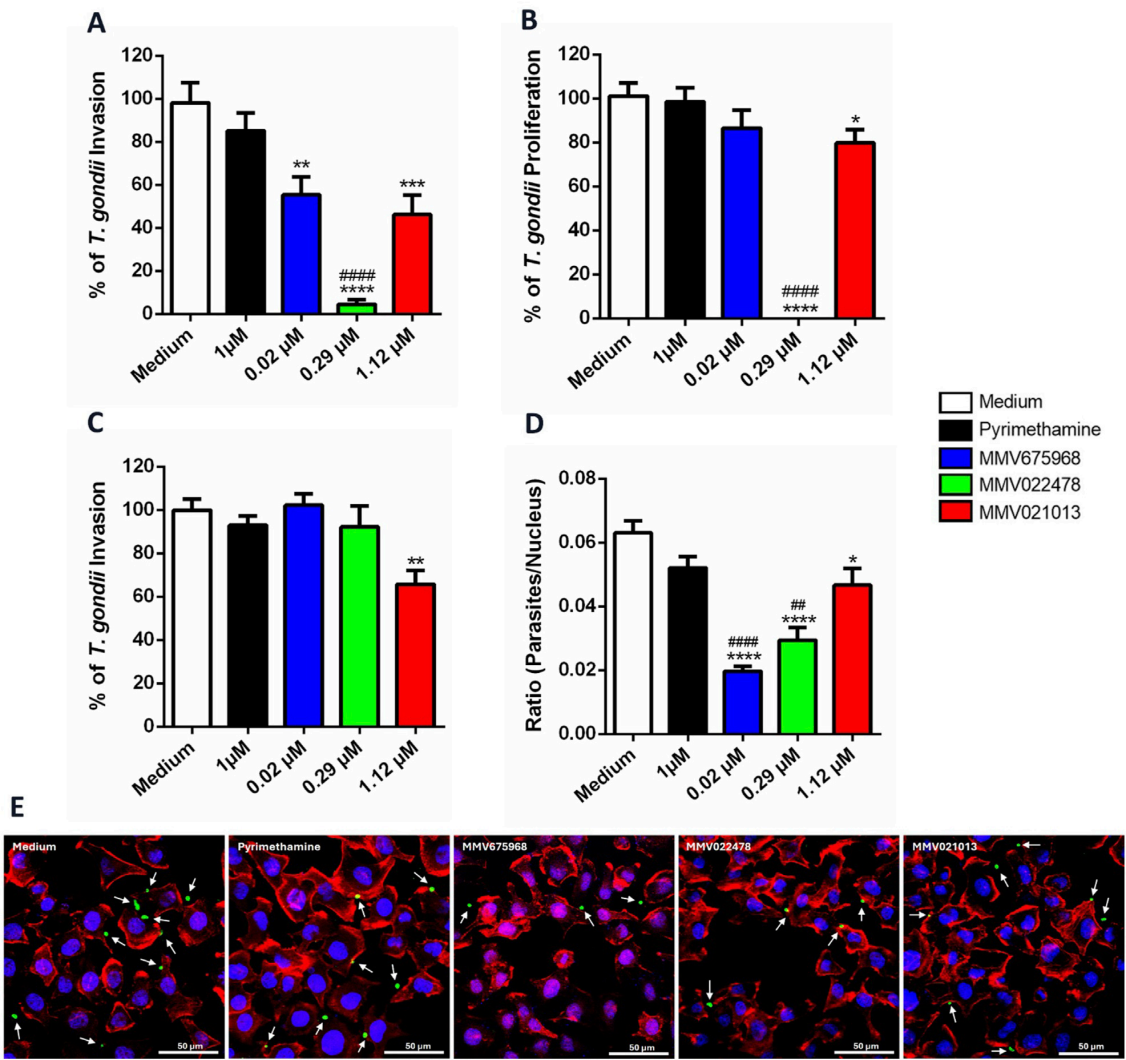
MMV675968, MMV022478, and MMV021013 interfere in early steps of *T. gondii* infection in BeWo cells. **(A,B)** *T. gondii* tachyzoites were pretreated for 1 h with MMV675968 (0.02 μM), MMV022478 (0.29 μM), MMV021013 (1.12 μM), PYR (1 μM), or culture medium only and then used to infect BeWo cells for 3 or 72 h to assess the infection and proliferation, respectively. The number of tachyzoites was quantified using the β-galactosidase assay and expressed as a percentage of *T. gondii* infection or proliferation. **(C)** BeWo cells were treated for 72 h with MMV675968 (0.02 μM), MMV022478 (0.29 μM), MMV021013 (1.12 μM), PYR (1 μM), or culture medium only and then were infected by *T. gondii* tachyzoites for 3 h. The number of tachyzoites was quantified using the β-galactosidase assay and expressed as a percentage of *T. gondii* infection. The results were normalized to the untreated control. **(D)** *T. gondii* tachyzoites were pretreated as described above and then allowed to interact with paraformaldehyde-fixed BeWo cells on glass coverslips for 3 h. Subsequently, cells and parasites were fixed and labeled with TO-PRO-3 Iodide (nuclei, blue), tetramethylrhodamine isothiocyanate (TRITC)-conjugated phalloidin (F-actin, red), and Alexa Fluor 488-conjugated anti-rabbit IgG (tachyzoites, green). Images were acquired using confocal fluorescence microscopy (Zeiss, LSM 510 Meta, Germany), with excitation wavelengths at 488 nm (Argon laser) for Alexa Fluor 488, 543 nm HeNe laser for TRITC, and 633 nm (HeNe laser) for TO-PRO-3. Emission detection wavelengths were set to 519 nm for Alexa Fluor 488, 570 nm for TRITC, and 661 nm for TO-PRO-3. Approximately 20 fields were examined randomly to obtain the number of cell nucleus and the total number of attached parasites per field analyzed. The results were expressed by the ratio (parasites/nucleus). **(E)** Representative fluorescence images are demonstrated, according to each experimental situation. Cells display typical trophoblastic morphology, including well-defined nuclei, a cobblestone-like arrangement, and intact cytoskeletal structure. White arrows indicate parasites adhered to the cells (scale bars: 50 μm). Data are shown as means ± standard error of the mean (SEM). ^*^ *p* < 0.05, ^**^ *p* < 0.01, ^***^ *p* < 0.001, and ^****^ *p* < 0.0001 indicate comparison between untreated and pre-treated parasites **(A,B,D)**; ^**^ *p* < 0.01indicates comparison between untreated and treated cells **(C)**; ^##^ *p* < 0.01 and ^####^ *p* < 0.0001 indicate comparison to PYR-treated parasites **(A,B,D)**; significant differences were analyzed using the one-way ANOVA test with Sidak’s multiple-comparison post hoc test. Differences were considered statistically significant when *p* < 0.05. Data are representative of at least three independent experiments, each performed with a minimum of eight replicates per group.

The results showed that exposure of tachyzoites to the compounds for 1 h, followed by 3 h of culture, resulted in a significant reduction in *T. gondii* infection rates compared to the control group (^**^ *p* < 0.01, MMV675968; ^****^ *p* < 0.0001, MMV022478; ^***^ *p* < 0.001, MMV021013; Figure 2A), suggesting that these treatments interfere with early steps of the host–parasite interaction. Notably, MMV022478 was more effective at controlling parasite infection than PYR (1 μM) (^####^ *p* < 0.0001; Figure 2A).

Regarding parasite proliferation, only pretreatment with MMV022478 (^****^ *p* < 0.0001; Figure 2B) and MMV021013 (^*^ *p* < 0.05) sustained antiparasitic activity, significantly inhibiting *T. gondii* proliferation compared to untreated parasites (Figure 2B). Furthermore, MMV022478 was significantly more effective than PYR in reducing parasite proliferation (^####^ *p* < 0.0001; Figure 2B). In contrast, pretreatment with PYR (1 μM) for 1 h did not significantly affect either *T. gondii* infection or intracellular proliferation (Figures 2A,B). To determine whether the compounds also act on host cells, we performed infection assays using pre-treated BeWo cells. Cells were exposed for 72 h to each compounds – MMV675968 (0.02 μM), MMV022478 (0.29 μM), or MMV021013 (1.12 μM) – prior to infection with *T. gondii* tachyzoites for 3 h. The β-galactosidase assay revealed that only MMV021013-treated cells showed a significantly reduced infection rate compared to the control group (^**^ *p* < 0.01; Figure 2C).

Given that treatment with MMV compounds reduced parasite infection in BeWo cells, we next evaluated their effect on parasite adhesion, an earlier stage of infection. *T. gondii* tachyzoites were pretreated for 1 h with MMV675968 (0.02 μM), MMV022478 (0.29 μM), and MMV021013 (1.12 μM), and then incubated for 3 h with BeWo cells previously fixed with 4% PFA. Parasites and host cells were stained, and slides were analyzed using confocal microscopy. Compared to the control group, all compounds significantly reduced parasite adhesion (^****^ *p* < 0.0001, MMV675968 and MMV022478; ^*^ *p* < 0.05, MMV021013; Figure 2D), as indicated by the calculated ratio (number of parasites/number of host cell nucleus). PYR (1 μM) treatment did not significantly affect parasite adhesion. Moreover, adhesion inhibition by MMV675968 (^####^ *p* < 0.0001) and MMV022478 (^##^ *p* < 0.01) was significantly greater than that observed with PYR (Figure 2D). Representative images illustrating the impact of treatments on parasite–host cell interaction are shown in Figure 2E.

### 3.3 Treatment with MMV675968, MMV022478, and MMV021013 induced damage to the ultrastructure of *T. gondii* tachyzoites

**FIGURE 3:**
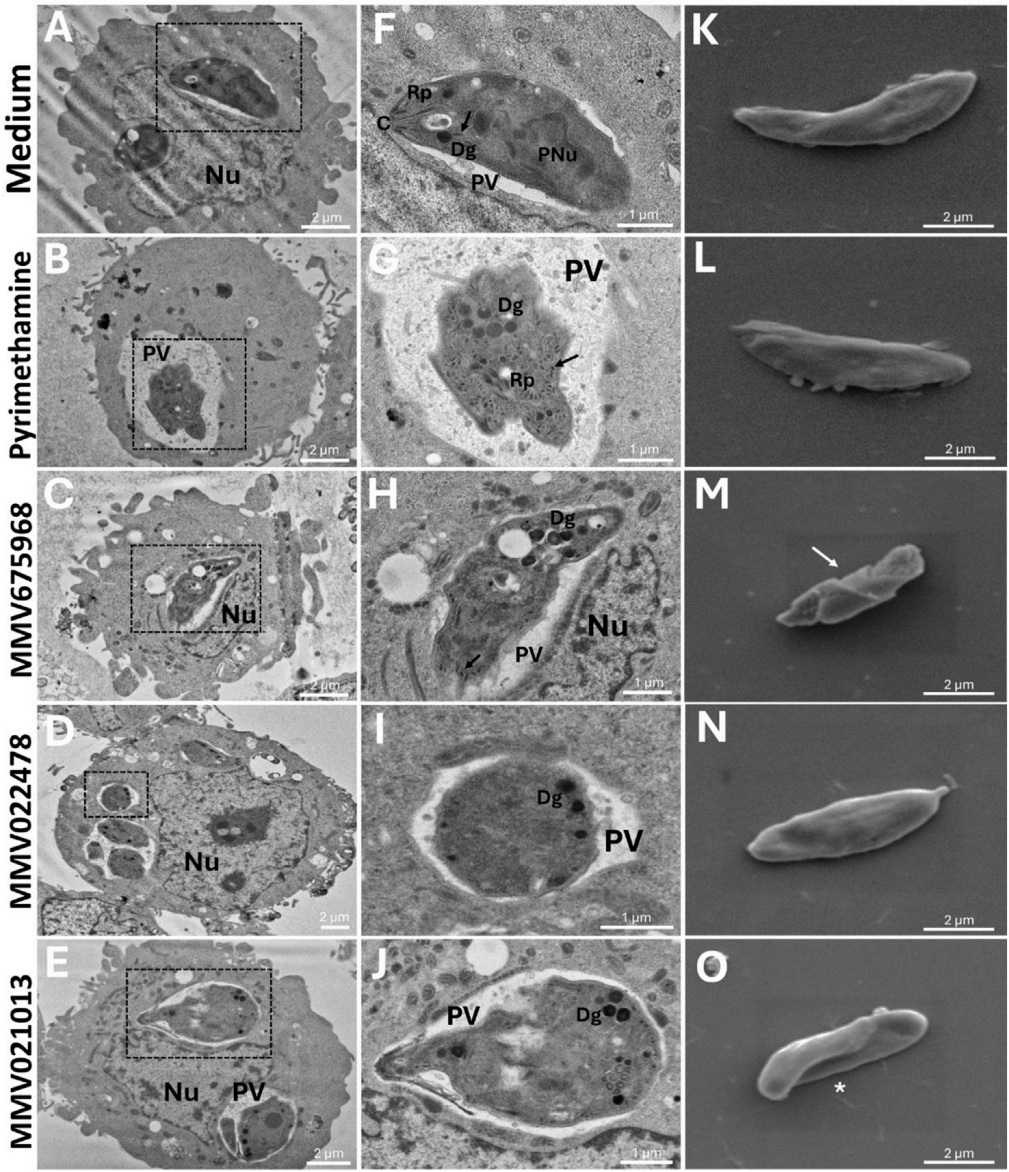
MMV675968, MMV022478, and MMV021013 induce morphological alterations in *T. gondii*. BeWo cells were infected with *T. gondii* tachyzoites (MOI of 1:1) for 3 h and incubated for 72 h with MMV675968 (0.02 μM), MMV022478 (0.29 μM), MMV021013 (1.12 μM), and PYR (1 μM) or culture medium only (untreated group). Transmission electron images were acquired at an accelerating voltage of 80 kV, with magnifications ranging from ×2,000 to ×6,000. Any brightness and contrast adjustments were applied uniformly across all samples to ensure accurate visualization of ultrastructural details. Representative micrographs are demonstrated, according to the experimental situation: **(A-F)** untreated cells, **(B-G)** PYR-treated cells, **(C-H)** MMV675968-treated cells, **(D-I)** MMV022478-treated cells, and **(E-J)** MMV021013-treated cells. Scale bars (bottom right): 1 and 2 μm. *T. gondii* tachyzoites were pre-treated for 1 h with MMV675968 (0.02 μM), MMV022478 (0.29 μM), MMV021013 (1.12 μM), PYR (1 μM), or culture medium only. Scanning electron images were acquired at an accelerating voltage of 5 kV, with magnification of ×10,000. Any brightness and contrast adjustments were applied uniformly across all images to ensure consistent representation of surface ultrastructure. Representative micrographs are demonstrated, according to the experimental situation: **(K)** untreated parasites, **(L)** PYR-treated parasites, **(M)** MMV675968-treated parasites, **(N)** MMV022478-treated parasites, **(O)** MMV021013-treated parasites (scale bars (bottom right): 2 μm). C, conoid; Dg, dense granule; Nu, host nucleus; PNu, parasite nucleus; PV, parasitophorous vacuole; Rp, rhoptries. Black arrows indicate micronemas. White arrows indicate ultrastructural alterations. White asterisks indicate torsion. Data are representative of at least three independent experiments, each performed with a minimum of eight replicates per group.

To further investigate the direct effects of the compounds on the parasite, *T. gondii*-infected BeWo cells were treated with the following: MMV675968 (0.02 μM), MMV022478 (0.29 μM), or MMV021013 (1.12 μM) for 72 h, and the ultrastructure of tachyzoites was analyzed using TEM. In the control group, no significant morphological alterations were observed. Parasites displayed well-defined boundaries, an intact pellicle, and partially identifiable organelles, including dense granules (Dg), micronemes (black arrows), rhoptries (Rp), and the conoid (C) (Figures 3A,F). In the PYR-treated group, tachyzoites exhibited severe abnormalities, including loss of typical shape, membrane deterioration, and organelle disorganization (Figures 3B,G). In the MMV675968-treated group, parasites presented irregular and ruptured membranes, along with cell body degradation, primarily due to the deterioration of organelles, which appeared poorly defined. Furthermore, vacuolization and accumulation of Dg were observed in the apical region, possibly indicating a stress response (Figures 3C,H). Treatment with MMV022478 resulted in marked alterations compared to the control group; tachyzoites exhibited a more rounded, swollen appearance and pronounced organelle disorganization (Figures 3D,I). In the MMV021013-treated group, parasites were also deformed, with an edematous and rounded morphology, clearly differing from the typical crescent shape of *T. gondii* tachyzoites. Organelle integrity was also compromised (Figures 3E,J). These findings indicate that all the three compounds from the Pathogen Box induced structural damage in *T. gondii* tachyzoites after 72 h of treatment.

Additionally, SEM was used to assess surface alterations following 1 h of pretreatment with the compounds. As shown in Figure 3K, untreated tachyzoites showed the characteristic crescent-shaped morphology, with no visible surface damage. Similarly, pretreatment with PYR (Figure 3L) or MMV022478 (Figure 3N) did not affect parasite morphology. In contrast, MMV675968 induced surface abnormalities such as membrane depressions, reduced body size, loss of symmetry, and collapse of surface structure (white arrow) (Figure 3M). Pretreatment with MMV021013 caused visible torsion of the parasite body (white asterisk) (Figure 3O). These results suggest that MMV675968 and MMV021013 can induce surface alterations in *T. gondii*, indicating a direct toxic effect that may compromise parasite viability.

Taken together, these results demonstrate that all three tested compounds can induce morphological damage in *T. gondii*, either after 72 h of treatment in infected cells or after a 1-h direct pretreatment. These structural alterations may underlie the observed impairment of the parasite’s ability to infect and survive within host cells.

### 3.4 MMV675968, MMV022478, and MMV021013 modulate cytokine levels in infected BeWo cells

**FIGURE 4:**
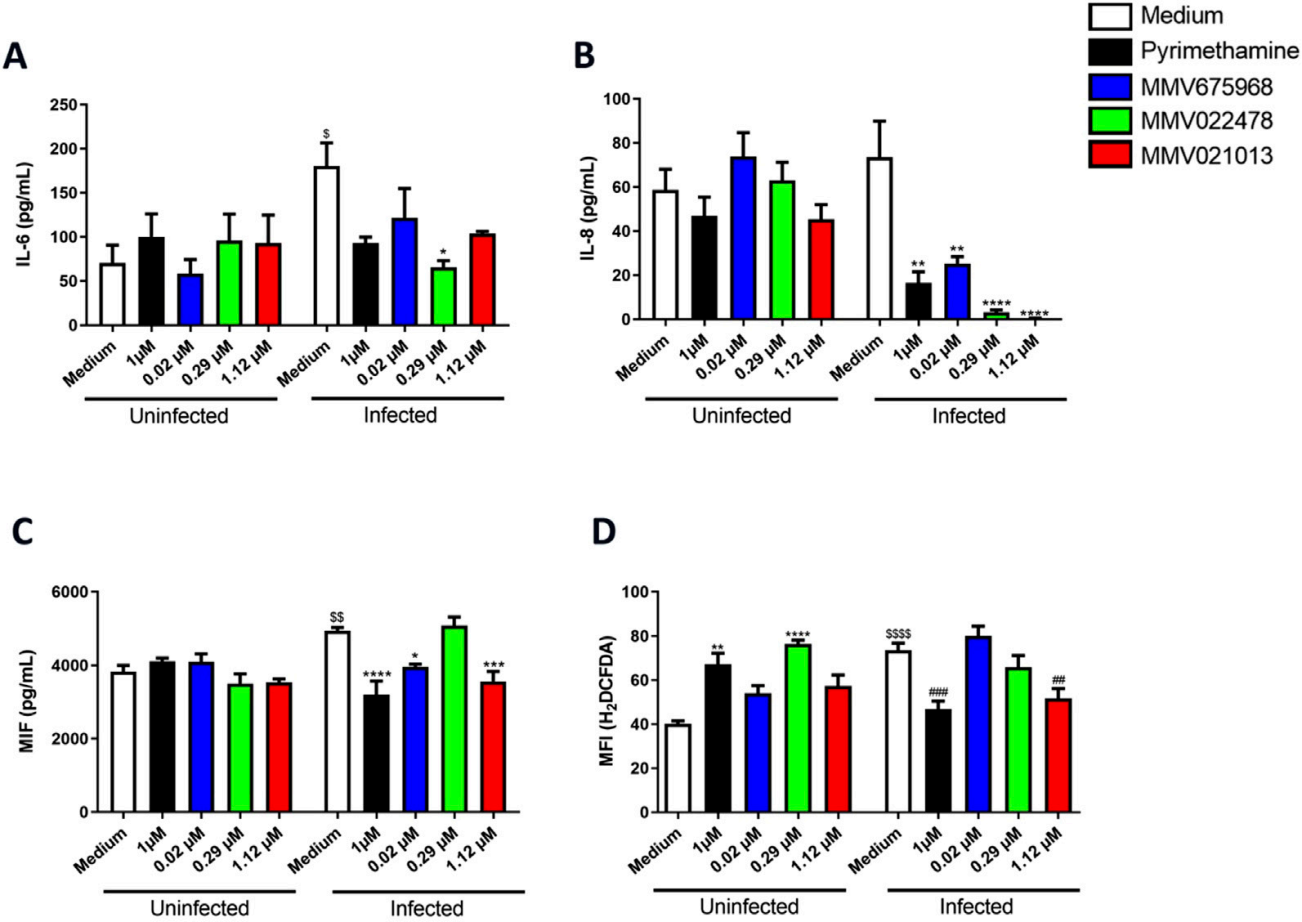
MMV675968, MMV022478, and MMV021013 decrease IL-8 and alter ROS production in BeWo cells. BeWo cells were infected or not with *T. gondii* tachyzoites, followed by treatment for 72 h with MMV675968 (0.02 μM), MMV022478 (0.29 μM), MMV021013 (1.12 μM), and PYR (1 μM) or culture medium only (untreated group). Cell culture supernatants were collected for measurement of **(A)** IL-6, **(B)** IL-8, and **(C)** MIF. Cytokine levels were expressed in pg/mL. **(D)** The fluorescent probe H_2_DCF-DA was used to measure the production of ROS in the BeWo cells. Data are shown as means ± standard error of the mean (SEM). ^*^ *p* < 0.05, ^**^ *p* < 0.01, ^***^ *p* < 0.001, and ^****^ *p* < 0.0001 indicate comparison between infected/untreated and infected/treated cell supernatants **(A,B,C)**; ^**^ *p* < 0.01 and ^****^ *p* < 0.0001 indicate comparison between uninfected/untreated and uninfected/treated cells **(D)**; ^$^ *p* < 0.05, ^$$^ *p* < 0.01, and ^$$$$^ *p* < 0.0001 indicate comparison between uninfected/untreated and infected/untreated supernatants/cells **(A,C,D)**; ^##^ *p* < 0.01 and ^###^ *p* < 0.001 indicate comparison between infected/untreated and infected/treated cells **(D)**. Significant differences were analyzed using the one-way ANOVA test with Sidak’s multiple-comparison post hoc test. Differences were considered statistically significant when *p* < 0.05. Data are representative of at least three independent experiments, each performed with a minimum of eight replicates per group.

To assess the immunomodulatory potential of the three selected Pathogen Box compounds in BeWo cells, the levels of cytokines IL-4, IL-6, IL-8, IL-10, TNF-α, and MIF were quantified. In uninfected cells, treatment with the compounds did not alter cytokine production compared to the untreated control (Figures 4A–C). On the other hand, *T. gondii* infection led to increased IL-6 and MIF levels in the infected/untreated group (^$^ *p* < 0.05 | ^$$^ *p* < 0.01, respectively; Figures 4A,C).

Treatment with both MMV675968 and MMV021013 significantly reduced IL-8 and MIF levels compared to the infected/untreated group (^**^ *p* < 0.01 | ^*^ *p* < 0.05 for MMV675968; ^****^ *p* < 0.0001 | ^***^ *p* < 0.001 for MMV021013, respectively; Figures 4B,C). Similarly, MMV022478 treatment attenuated IL-6 and IL-8 production (^*^ *p* < 0.05 | ^****^ *p* < 0.0001, respectively; Figures 4A,B). PYR treatment also led to reduced IL-8 and MIF levels in infected cells (^**^ *p* < 0.01 | ^***^ *p* < 0.0001, respectively; Figures 4B,C), whereas it did not alter cytokine production in uninfected cells. IL-4, IL-10, and TNF-α levels were below the detection limit in all experimental conditions, regardless of infection status (data not shown).

We also evaluated whether the compounds influenced ROS production. In the absence of infection, cells treated with PYR and MMV022478 exhibited increased ROS levels compared to the uninfected/untreated control (^**^ *p* < 0.0001 | ^****^ *p* < 0.01, respectively; Figure 4D). *T. gondii* infection alone significantly increased ROS levels relative to the uninfected group (^$$$$^ *p* < 0.0001). However, infected cells treated with PYR or MMV021013 showed a significant reduction in ROS levels compared to the infected/untreated control (^###^ *p* < 0.01 | ^##^ *p* < 0.001, respectively; Figure 4D).

Together, these findings suggest that the evaluated Pathogen Box compounds exert immunomodulatory effects in *T. gondii*-infected BeWo cells, primarily by reducing IL-8 and MIF production. Additionally, they can modulate oxidative stress by regulating ROS levels.

### 3.5 MMV675968, MMV022478, and MMV021013 control *T. gondii* proliferation in human placental explants

**FIGURE 5:**
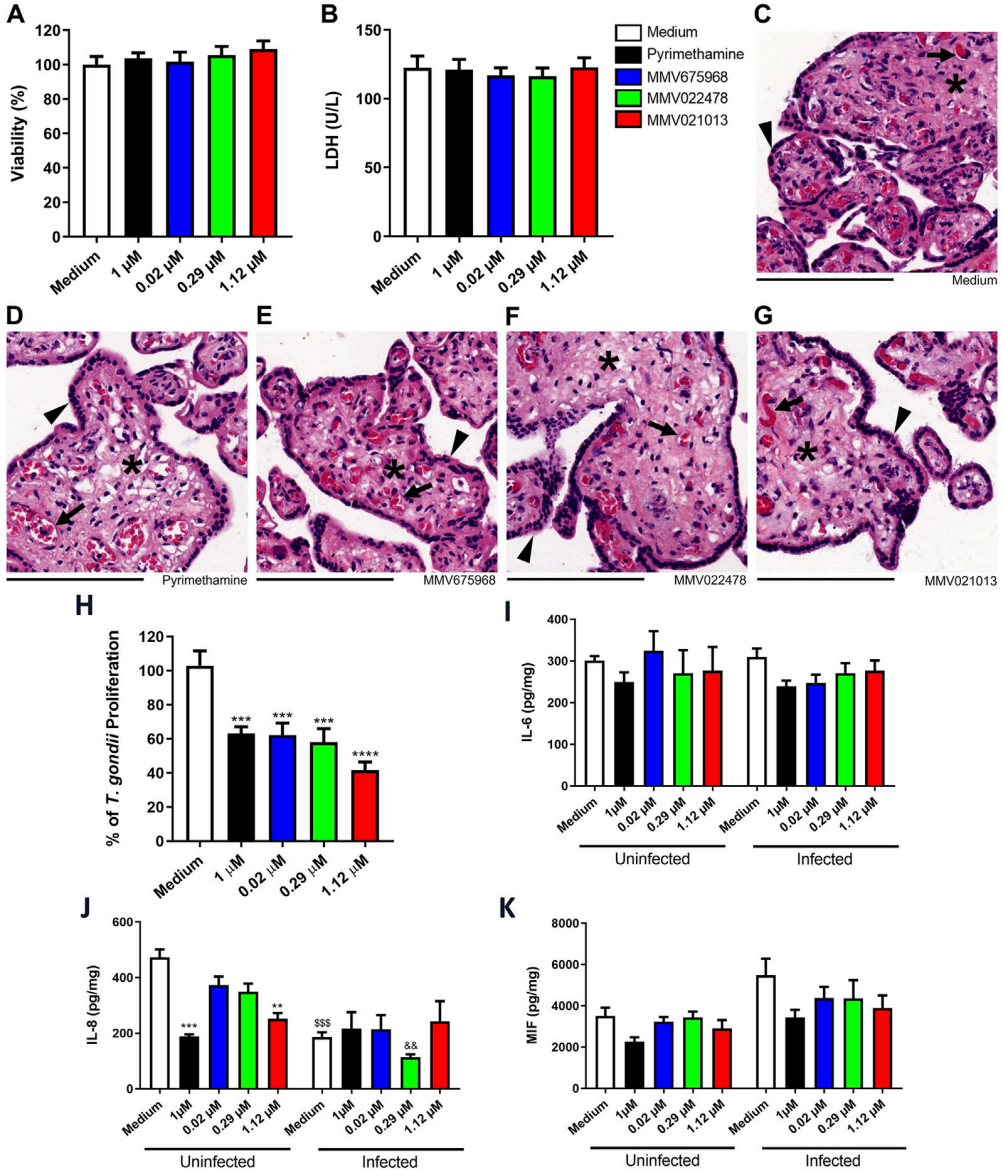
MMV675968, MMV022478, and MMV021013 inhibit *T. gondii* proliferation in human placental explants. Villous explants were incubated for 72 h with MMV675968 (0.02 μM), MMV022478 (0.29 μM), MMV021013 (1.12 μM), and PYR (1 μM) or culture medium only (untreated group) and subsequently assessed for viability. **(A)** The viability of villi was assessed using the MTT assay and expressed as the mean percentage (%) of viable villi. Untreated explants served as the positive control for the viability of villi and were considered 100% viable. The results were normalized to the untreated control. **(B)** Collected supernatants of villi were analyzed to determine LDH levels (U/L). Histological sections were stained with hematoxylin–eosin (HE). Representative fields, at ×10 of magnification with 200 µm scale bar, are demonstrated, according to the experimental situation: **(C)** untreated villi, **(D)** PYR-treated villi, **(E)** MMV675968-treated villi, **(F)** MMV022478-treated villi, and **(G)** MMV021013-treated. **(H)** Villous explants were infected with *T. gondii* tachyzoites for 24 h, followed by treatment for additional 72 h with either MMV675968 (0.02 μM), MMV022478 (0.29 μM), MMV021013 (1.12 μM), and PYR (1 μM) or culture medium only (untreated group). Intracellular parasite proliferation was assessed using the β-galactosidase assay and represented as percentage *T. gondii* proliferation, with the infected/untreated group (control) considered 100% of parasite proliferation. The supernatants from uninfected or infected villi after treatments were collected for measurement of **(I)** IL-6, **(J)** IL-8, and **(K)** MIF. Cytokine levels were expressed in pg/mg of tissue. Data are shown as means ± standard error of the mean (SEM). ^***^ *p* < 0.001 and ^****^ *p* < 0.0001 indicate comparison between untreated and treated villi **(H)**; ^**^ *p* < 0.01 and ^***^ *p* < 0.001 indicate comparison between uninfected/untreated and uninfected/treated supernatants **(J)**; ^$$$^ *p* < 0.001 indicates comparison between uninfected/untreated and infected/untreated supernatants **(J)**. Significant differences were analyzed using the one-way ANOVA test with Sidak’s multiple comparison *post hoc* test. Differences were considered statistically significant when *p* < 0.05. Arrowheads indicate the outer multinucleated syncytiotrophoblast layer. Asterisks indicate the cellularized mesenchymal tissue, externally covered by the syncytiotrophoblast and inside filling the placental explants’ structure. Black arrows indicate mesenchyme-surrounded fetal blood vessels, which contains preserved fetal blood cells, mostly fetal erythrocytes with typical eosinophilia. Data are representative of at least three independent experiments, each performed with a minimum of eight replicates per group.

*Ex vivo* models of human placental explants are widely used to study *T. gondii* infection at the maternal–fetal interface. Before conducting antiparasitic assays, we assessed the viability of human placental explants exposed to the compounds—at the same concentrations used in BeWo cells—for 72 h, using MTT and LDH assays. The results showed that none of the tested compounds, including the reference drug PYR, were toxic to the placental villi compared to the untreated control (Figures 5A,B).

To corroborate biochemical data, we performed hematoxylin–eosin (HE) staining to analyze the morphology and structural integrity of the chorionic villi. Explants treated with the compounds or PYR exhibited morphological features similar to those of the untreated control, including a well-preserved outer multinucleated syncytiotrophoblast layer (arrowheads) covering the cellularized mesenchymal core (asterisks), which contains intact fetal blood cells (black arrows) (Figures 5C–G).

After confirming the absence of cytotoxicity, we evaluated the ability of the compounds to control *T. gondii* proliferation in placental tissue using the β-galactosidase assay. After 72 h of treatment, all three compounds, along with PYR, significantly reduced parasite proliferation compared to the untreated control (^***^ *p* < 0.001, PYR, MMV675968, and MMV022478 | ^****^ *p* < 0.0001, MMV021013; Figure 5H).

We also evaluated cytokine secretion in the supernatants of placental explant cultures under distinct experimental conditions. Overall, IL-6 and MIF levels did not show significant changes, regardless of *T. gondii* infection (Figures 5I,K). However, both PYR and MMV021013 reduced IL-8 release in uninfected explants compared to the uninfected/untreated control (^***^ *p* < 0.001 | ^**^ *p* < 0.01, respectively; Figure 5J). Additionally, *T. gondii* infection alone reduced IL-8 production compared to the uninfected/untreated group (^$$$^ *p* < 0.001; Figure 5J) although the compounds did not further modulate IL-8 levels in infected BeWo cells.

IL-4, IL-10, and TNF remained below the detection limit in all experimental groups, regardless of infection status (data not shown).

### 3.6 MMV021013 exhibits the most favorable ADMET profile among the evaluated MMV compounds

**FIGURE 6:**
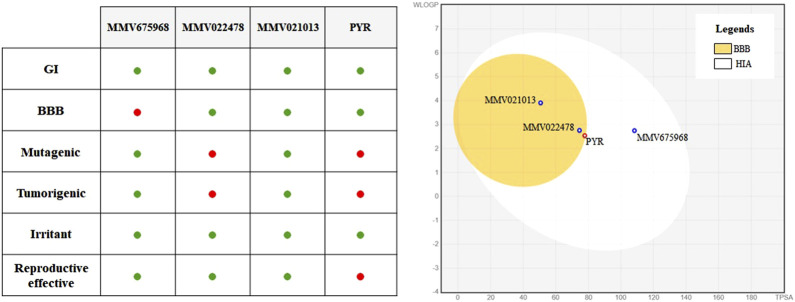
*In silico* ADMET profiling of selected MMV compounds and pyrimethamine (PYR). On the left, a summary table highlights key ADMET properties, with favorable outcomes represented by green dots and unfavorable outcomes by red dots. MMV021013 stands out for presenting high gastrointestinal absorption without predicted toxicity and blood–brain barrier (BBB) permeability, indicating a safer profile than that of PYR. On the right, the BOILED-Egg diagram predicts human gastrointestinal absorption (HIA) and BBB permeability.

ADMET profiling was performed to evaluate the pharmacokinetic and toxicity-related characteristics of the selected compounds and PYR (Figure 6). All compounds, including PYR, were predicted to have high GI absorption, suggesting favorable oral bioavailability. Regarding BBB permeability, MMV022478, MMV021013, and PYR were predicted to be BBB permeant, whereas MMV675968 was not. Concerning toxicity parameters, MMV675968 and MMV021013 showed the most favorable profiles, with no predicted mutagenic, tumorigenic, irritant, or reproductive toxicity. MMV022478 and PYR, however, presented a higher toxicity risk. Both were predicted to be mutagenic and tumorigenic, and PYR was additionally predicted to have reproductive toxicity.

## 4 Discussion

The drugs currently available for the treatment of congenital toxoplasmosis present several limitations, making this disease a serious global health concern. Consequently, the search for alternative therapeutic strategies that are both effective and safe is increasingly necessary. In this context, the MMV offers a diverse collection of synthetic compounds for drug repurposing, an approach that has been extensively explored in recent years to identify new treatments for infections caused by various pathogens (Wang et al., 2019; Maher et al., 2023; Tyagi et al., 2023; Costa et al., 2024).

Spalenka et al. (2018) screened 400 compounds from MMV’s Pathogen Box and identified several candidates with anti-*T. gondii* activity. Among them, compounds were selected based on the following: low toxicity to Vero cells, an IC_50_ below 2 μM, and an SI above 4. Despite their promising activity, there are no reports on the evaluation of these compounds in models relevant to congenital toxoplasmosis. In this study, we investigated the efficacy of three Pathogen Box compounds—MMV675968, MMV022478, and MMV021013—in controlling *T. gondii* infection in human trophoblastic cells (BeWo) and in third-trimester human placental villi.

We first evaluated antiparasitic efficacy and cytotoxicity at three concentrations: the IC_50_ reported by Spalenka et al. (2018), along with half and double that value. The compounds showed minimal cytotoxicity after 72 h, except for MMV021013 at its highest concentration. All three compounds significantly reduced *T. gondii* proliferation in BeWo cells, with some concentrations (MMV675968 at 0.04 μM, MMV022478 at 0.29 and 0.58 μM, and MMV021013 at 2.24 μM) outperforming PYR. Notably, MMV022478 and MMV021013, along with PYR, sustained their antiproliferative effects 24 h after withdrawing treatment, suggesting a long-lasting impact.

To investigate potential mechanisms of action, we conducted experiments using pretreatment assays on either the parasite or the cells. MMV675968 pretreatment reduced infection but not proliferation, suggesting that longer exposure or higher concentrations are required for full activity. In contrast, MMV022478 was highly effective after only 1 h of parasite exposure, significantly impairing both infection and replication. MMV021013 affected the infection, suggesting a dual action on the host and parasite. All three compounds reduced parasite adhesion, with MMV675968 being the most potent. PYR, although effective in reducing parasite replication in infected BeWo cells, did not impair adhesion, infection, or proliferation when used as a 1-h pretreatment. These results suggest that the Pathogen Box compounds interfere with multiple steps of the *T. gondii* lytic cycle.

The observation that PYR irreversibly inhibits parasite replication only after internalization is consistent with its known mechanism—dihydrofolate reductase (DHFR) inhibition (Veale, 2019). MMV675968, a quinazoline-2,4-diamine, is also a known DHFR inhibitor. Previous studies have shown its activity against DHFR in *Acinetobacter baumannii* and *Streptococcus suis* (Songsungthong et al., 2019; Songsungthong et al., 2021). Additionally, *in silico* docking studies indicate that MMV675968 interacts with bacterial DHFR but not with the human enzyme, disrupting folate biosynthesis and parasite replication; however, the compound may also act on other cellular processes, such as adhesion and infection (Sharma et al., 2023). The inhibition of DHFR is a well-established therapeutic target for the treatment of toxoplasmosis. Studies have shown that silencing the DHFR-TS gene in *T. gondii* results in decreased parasite viability and increased survival time of infected mice (Azimi-Resketi et al., 2020). Furthermore, the discovery of selective DHFR inhibitors for *T. gondii* highlights the importance of this target in antiparasitic therapy (Hopper et al., 2019). It is important to mention that MMV675968 also stands out as a potent inhibitor of late stages in *Plasmodium falciparum* development, particularly in blocking the ring–trophozoite transition, with submicromolar EC_50_ values, which suggests that this compound may act via mechanisms conserved among apicomplexans (Patra et al., 2020).

MMV022478 is a pyrazolopyrimidine associated with the inhibition of protein kinase C (PKC) and suppression of NADPH oxidase complex formation (Borbély et al., 2010; Altenhöfer et al., 2015). Previous studies have shown that PKC modulation is involved in *T. gondii* intracellular survival. *T. gondii*’s PKC interacts with host autophagy proteins, such as Atg3 and Atg5, to promote parasite intracellular survival, indicating that PKC-mediated signaling is critical for parasite survival within the host cell (Wu et al., 2022). Some studies also suggest that it may target DHFR due to structural similarities with PYR (Secrieru et al., 2020). López-Arencibia et al. (2021) observed that MMV022478 induced ROS accumulation and autophagy in *Leishmania* (*Leishmania*) *amazonensis*. Our findings are consistent with these studies, highlighting its broad antiparasitic activity. Although further investigation is needed, it is likely that this drug’s mechanism of action against *T. gondii* involves inhibition of either protein kinase C—thus preventing NADPH oxidase complex assembly—or the DHFR enzyme.

MMV021013, a 2-pyridyl-4-aminopyrimidine, is active against *Leishmania donovani*, *Trypanosoma cruzi* (Duffy et al., 2017), and fungi (Coelho et al., 2020). It is known to inhibit methionine aminopeptidases, which are enzymes essential for apicoplast biogenesis in *T. gondii* (Zheng et al., 2020). Other aminopeptidases, such as M17 leucine aminopeptidase, are also important for *T. gondii* virulence (Que et al., 2007; Zheng et al., 2015). In this manner, protease inhibition plays a crucial role in parasite viability. The serine protease protein TgPI-1 protects *T. gondii* against proteases in the gastrointestinal tract and neutrophil-derived proteins in the lamina propria, suggesting that the regulation of proteolytic activity is essential for intracellular survival (Saffarian et al., 2025). The effects of MMV021013 on parasite adhesion, infection, and replication are consistent with these biological roles, suggesting that it may act through protease inhibition.

Ultrastructural analysis confirmed direct parasite damage following treatment with all three compounds. Observed alterations, including membrane rupture, cytoplasmic disorganization, abnormal morphology, and vacuolization, support the conclusion that these compounds compromise *T. gondii* viability. The greater extent of damage following prolonged exposure suggests that intracellular accumulation contributes to full cytotoxic effects although MMV675968 and MMV021013 also caused changes after only 1 h, indicating a rapid onset of action.

Although some vacuolization processes can occur in reversible stress responses, our reversibility assay indicates that the morphological alterations observed in TEM data are more consistent with terminal damage, once the lack of recovery following drug withdrawal supports the interpretation of irreversible injury. The ultrastructural changes observed in our samples may be associated with autophagy-like processes. This phenotype has been widely described in protozoa exposed to stress conditions or different drugs, in which electron microscopy revealed an increased number of autophagosomes, the formation of myelin-like structures, and endoplasmic reticulum profiles surrounding organelles (Pedra-Rezende et al., 2022). In such cases, the exacerbated activation of autophagic pathways has been linked not only to the loss of cellular homeostasis but also to reduced virulence and impaired differentiation capacity of the parasites. In this context, although further functional assays would be required to confirm autophagy in our model, the decreased proliferation observed in our assays may be related to this process, suggesting that the induction of autophagy-like phenotypes could represent a potential mechanism for controlling pathogenicity.

In addition, our findings are in line with recent drug repurposing efforts for *T. gondii*. Dos Santos M. et al. (2023b) screened the MMV’s Pandemic Box and identified compounds highly active against tachyzoites, also reporting ultrastructural alterations by electron microscopy similar to those observed here, underscoring the ability of drug candidates to interfere with organelle integrity and parasite replication. These parallels highlight that diverse chemical scaffolds, originally developed for other diseases, may converge on comparable cellular targets or pathways in *T. gondii*.

The ultrastructural alterations observed in our study may arise either from direct cytotoxic effects of the tested compounds on *T. gondii* or from secondary host cell responses. Although our experimental design was not intended to dissect these mechanisms, the use of an *in vitro* model and the reproducibility of the ultrastructural changes across independent replicates strongly suggest that the primary factor is the direct action of the compounds on the parasite. Nevertheless, it cannot be excluded that host cell responses may also contribute to the observed phenotypes, particularly given the complex host–parasite interactions that influence intracellular survival. Further studies particularly designed to separate direct parasitic from host-mediated effects will be required to fully address this distinction.

Given the importance of host immune responses at the maternal–fetal interface, we evaluated cytokine profiles in infected BeWo cells. Infection increased IL-6 and MIF levels. Treatment with the three compounds reduced IL-8 and, in some cases, IL-6 or MIF. PYR also reduced IL-8 and MIF. IL-6 and MIF are typically elevated during infection and contribute to inflammation, but our findings suggest that their reduction does not impair parasite control in BeWo cells. IL-8, which has been associated with parasite dissemination, was significantly reduced by all three compounds (Sommerville et al., 2013). Previous studies have linked decreased IL-8 to parasite control in trophoblastic cells (Martínez et al., 2023; Teixeira et al., 2024; Teixeira et al., 2025). Thus, we proposed that the anti-*T. gondii* effects of these compounds may involve IL-8 suppression, contributing to reduced dissemination and parasite load.

We also evaluated the modulation of oxidative stress. In uninfected cells, MMV022478 and PYR increased ROS, whereas in infected cells, MMV021013 and PYR reduced ROS production. This suggests that the compounds can promote infection control without relying on oxidative stress pathways, reinforcing the hypothesis that their effects involve direct antiparasitic action.

In human placental explants, none of the compounds exhibited cytotoxicity, and all were effective in reducing parasite proliferation. Interestingly, cytokine production in placental explants was less responsive to treatment than that in BeWo cells. *T. gondii* infection reduced IL-8 but did not alter IL-6 of MIF levels. Treatment did not modulate cytokines in infected explants, suggesting that other mechanisms may be responsible for parasite control in this tissue model.

PYR effectively controlled infection in both *in vitro* and *ex vivo* models but lacked activity in early infection steps, unlike the Pathogen Box compounds. Based on the results presented here, we propose that MMV675968 may inhibit *T. gondii* DHFR, MMV022478 may act via DHFR or PKC inhibition, and MMV021013 may inhibit aminopeptidases. These compounds also promoted parasite structural damage and reduced IL-8 production in BeWo cells, supporting their potential as alternative therapies for congenital toxoplasmosis.

*In silico* prediction of ADMET properties is a valuable tool in the early stages of drug development as it helps anticipate pharmacokinetic behavior and potential safety issues. Among the compounds evaluated, both MMV022478 and PYR were predicted to cross the BBB and to present mutagenic and tumorigenic risks, with PYR also showing a predicted potential for reproductive toxicity. These results are consistent with previously reported adverse effects associated with PYR use during pregnancy, including bone marrow suppression and teratogenicity. In contrast, MMV021013 demonstrated predicted permeability through the BBB, without signs of mutagenicity or reproductive toxicity. Considering that *T. gondii* is known to form persistent cysts within brain tissue, BBB penetration is a critical feature for achieving therapeutic efficacy in the central nervous system. Moreover, it is reasonable to hypothesize that compounds with BBB permeability may also have the capacity to cross the placental barrier, as both barriers share several physicochemical and transporter-related characteristics. Lipophilic compounds of low molecular weight, particularly those not subjected to significant efflux by placental transporters, often diffuse across the placental interface. Thus, MMV021013 emerges as a particularly promising candidate for treating congenital toxoplasmosis as it may effectively reach both the fetal compartment and the brain, two key targets in the pathogenesis of the disease. Further studies are warranted to directly assess its placental permeability and *in vivo* distribution.

To complement the *in silico* ADME predictions, we also considered the physicochemical properties of the compounds, particularly molecular weight (MW) and lipophilicity, as these parameters strongly influence absorption, distribution, and tissue penetration. MMV675968 (MW = 359.81 g/mol; consensus LogP = 2.46), MMV022478 (MW = 432.91 g/mol; consensus LogP = 2.92), and MMV021013 (MW = 294.39 g/mol; consensus LogP = 3.40) fall within the ranges generally considered favorable for oral bioavailability. Nevertheless, it is important to emphasize that these values are computational predictions and may not fully recapitulate the *in vivo* pharmacokinetic behavior. Therefore, experimental validation of ADME parameters, including permeability assays and pharmacokinetic studies in animal models, will be essential to confirm the translational potential of these compounds.

A limitation of the present study is the exclusive use of *in vitro* and *ex vivo* models, which, although informative, cannot fully recapitulate the complexity of the *in vivo* environment. As such, further *in vivo* studies will be essential to validate the therapeutic potential of the tested compounds and to better understand their effects within a physiological context. Another limitation of the present work is that only short-term exposure was evaluated, which enabled the identification of early morphological and functional alterations in the parasite. However, this time frame does not provide information on long-term efficacy or the potential development of resistance, both of which represent crucial aspects for therapeutic translation. Subsequent studies should, therefore, be designed to address these questions, extending treatment periods and incorporating assays to monitor resistance mechanisms. Regarding the *ex vivo* assays, all placentas were collected following the same criteria: full-term, from cesarean deliveries, and from donors without comorbidities, ensuring a standardized sample. Despite this, variability between donors can still influence the observed outcomes.

## 5 Conclusion

In conclusion, our findings demonstrate that the Pathogen Box compounds—MMV675968, MMV022478, and MMV021013—exhibit distinct yet effective mechanisms of action against *T. gondii*, targeting key stages of the parasite’s lytic cycle, including adhesion, infection, and proliferation. These compounds exhibit broader activity profiles than PYR, making them promising candidates for alternative therapies in the treatment of vertically transmitted toxoplasmosis.

In addition, *in silico* ADMET predictions revealed that MMV021013 stands out as the most promising compound, combining high gastrointestinal absorption, blood–brain barrier permeability, and a favorable toxicity profile, with no predicted mutagenic, tumorigenic, irritant, or reproductive effects. This is particularly relevant in the context of congenital toxoplasmosis, where both placental and cerebral tissues are critical sites of infection.

Future investigations should aim to elucidate the specific molecular targets of these compounds in *T. gondii* and to evaluate their efficacy and safety in *in vivo* models of congenital toxoplasmosis. Moreover, investigating combinations of the tested compounds—or their use in association with established therapies—may offer synergistic effects and broaden the range of effective treatment strategies for toxoplasmosis.

## Data Availability

The raw data supporting the conclusions of this article will be made available by the authors, without undue reservation.
